# Some Issues with Statistical Crystal Plasticity Models: Description of the Effects Triggered in FCC Crystals by Loading with Strain-Path Changes

**DOI:** 10.3390/ma15196586

**Published:** 2022-09-22

**Authors:** Alexey Shveykin, Kirill Romanov, Peter Trusov

**Affiliations:** Laboratory of Multilevel Structural and Functional Materials Modeling, Perm National Research Polytechnic University, 614990 Perm, Russia

**Keywords:** crystal plasticity, two-level statistical constitutive model, complex loading, strain-path changes, yield surface

## Abstract

The justification of the applicability of constitutive models to exploring technological processes requires a detailed analysis of their performance when they are used to describe loadings including the complex loading mode that is characteristic of these processes. This paper considers the effect of equivalent stress overshooting after the strain-path changes known to occur in metals and alloys. The macrophenomenological and multilevel models, which are based on crystal plasticity, account for this effect by applying anisotropic yield criteria at the macro- and mesolevels, respectively. We introduce a two-level constitutive statistical inelastic deformation model (identified for aluminum) that incorporates the popular simple phenomenological anisotropic hardening law for describing the behavior of FCC polycrystals. The results of the numerical simulation are in satisfactory agreement with existing experimental data. Statistical analysis of the motion of a mesostress in the stress space on the crystallite yield surface is performed. The obtained data are compared with the results found using the isotropic hardening law. The results clarify the simulation details of statistical crystal plasticity models under loading with strain-path changes in materials and demonstrate their suitability for describing the processes under consideration.

## 1. Introduction

The use of empirical methods for the research and development of the technological processes of the thermomechanical treatment of materials and alloys [1,2,3] is very costly in terms of time and economic resources, especially due to the problems associated with the determination of processing regimes for advanced materials (functional materials—products) [4]. Therefore, one of the most important tasks faced by researchers is to develop mathematical models for these processes. In constructing the models, constitutive relations or constitutive models play a key role [5,6,7]; they should be able to account for changes in the structures of different materials. It has been established that the effective (operational) properties of materials significantly depend on their structures [8].

In recent years, much attention has been given to constructing multilevel constitutive models (CMs) of materials within the crystal plasticity (CP) framework [4,9,10,11,12,13,14,15,16,17,18]. Such CMs are based on the introduction of internal variables (IVs) that enables one to explicitly describe the meso- and microstructures of the materials and the deformation mechanisms and their “carriers” at various scale levels. The evolution of IVs is described in the framework of these CMs using kinetic equations highlighting the changes in the structures of materials and their effective physical and mechanical properties [6,19,20,21]. The advantages of multilevel CMs are related to their great versatility. The physical mechanisms that control the material behavior are similar, and therefore the structures of CMs are almost identical for the major classes of materials [6]. Certainly, the application of CMs to exploring various materials requires the identification of a number of parameters included in the model formulation; however, some of these are the well-known parameters contained in reference books.

When designing advanced CP models, for example, those that describe grain boundary sliding [22], it is important to make sure that the basic model is correct. For this purpose, apart from the theoretical analysis of the relations used in the model [23], the model itself should be carefully verified by assessing the results obtained by the basic CMs (with the same parameters) for various types of loadings, and not just for monotonic loading generally applied for parameter identification. This is why special attention should be paid to complex (at the macrolevel) loadings with a strain-path change (SPC) [24,25,26,27,28], which are inherent in real metal-forming processes. For example, during deep drawing, at a certain stage, there is a change in the strain mode from pure shear to biaxial tension [29]. An abrupt change in the strain path occurs in the equal-channel angular pressing (ECAP) process when a sample is rotated between successive passes [30]. Thus, the multilevel CMs should grant researchers the ability to describe the effects detected in complex loading experiments. It should be noted that the effects triggered by complex loading are most commonly investigated in experiments that involve SPCs and are performed on tubular specimens where each of the two sections of strain is realized at monotonic (proportional) loading with unloading between the loading stages or in a continuous manner. It is these loadings that will be considered in our study.

As is known, in most cases that occur during active complex loading immediately after the SPC, the equivalent stress σ_e_ decreases (the stress “dive” effect) on the stress–strain curve illustrating the dependence of σ_e_ on the equivalent strain ε_e_, and then the equivalent stress tends (usually smoothly) to return to the values corresponding to monotonic loading [24,25,31,32,33,34,35]. However, in other cases, after the stress “dive” effect, it sharply increases, exceeding the value preceding the SPC [17,31]. Sometimes, in particular when the SPC takes place, the σ_e_–ε_e_ curve, after a certain strain, matches a similar curve that corresponds to monotonic loading at the second strain path. This can be interpreted as a manifestation of the material memory time delay effect [5,36,37] (or delaying of scalar and vector material properties [38]). For the sake of brevity, the achievement of reduced and increased equivalent stresses after the SPC is called the stress–strain curve monotonicity change effect. The effect of SPCs on the response of aluminum and its alloys has been considered in numerous works, e.g., in [39,40,41,42,43,44,45].

It is interesting that for some materials, after the SPC, the values of the σ_e_–ε_e_ curve exceed the values of σ_e_ of a similar curve constructed for monotonic loading in the strain mode of the second section. This behavior is called the “cross-hardening effect” [46,47,48]. There are many experimental confirmations of the cross-hardening effect for various materials and loading modes: commercially pure copper [49,50], aluminum and its alloys [51,52,53,54], and steels [51,53,55,56]. It is worth noting that these full-scale experiments were carried out not under active loading but with unloading between stages. The point is that continuous loadings of the types under consideration, in particular rolling and tension sequences, cannot be implemented experimentally.

In some experimental studies [48,51,53], the cross-hardening effect was investigated by analyzing the interactions between the dislocations. In the first stage, a certain dislocation substructure is formed under loading. When the strain path changes, the dislocations begin to move along new slip systems (SSs), which can cross the structural formations created in the first stage. To overcome these formations, greater stresses are needed in comparison with those triggered by monotonic loading. It was shown in [54] that for metals with a low stacking fault energy (SFE), the effect of a change in the equivalent stress due to the SPC was less pronounced than for metals with a high stacking fault energy. This can be attributed to the fact that in low-SFE materials, the interactions between dislocations of active and inactive SSs result in the formation of strong barriers of the Lomer–Cottrell type, which equally strengthens both active and inactive SSs.

In macrophenomenological models, the cross-hardening effect is taken into account by specifying the evolution of the yield surface (YS). Basically, two groups of models are used. In the first group, transient processes are modeled by the shifting, expanding, or shrinking of the yield surface with its shape remaining unchanged. One of the first models developed in the framework of this approach was proposed in [57]. In [58], the authors presented a plasticity model that involved tensors to highlight the strain history. Later, this model was modified to describe softening [54], and was given the name “the MHH model”. The second group includes models that contain relations capable of taking into account the shape changes of the yield surface [59]. In [60], as an alternative to kinematic hardening, the model for describing a complex shape change of the yield surface (the HAH model) was developed and then extended to describe the orthogonal hardening and softening effects [56,61,62,63]. A common feature of the proposed macrophenomenological models is the need for a complex formulation of the evolution equations for the yield surface to better approximate the experimental data for the individual loading cases. So, we can conclude that these models are not universal because they cannot be applied to describe other materials and other conditions. This is also true for the macrophenomenological models that are based on the theory of elastoplastic processes [37,38] and the endochronic theory of plasticity [64,65].

There are many studies [47,52,66,67,68,69,70] where the cross-hardening effect has been described in terms of multilevel CP CMs that involve the laws of anisotropic hardening for various SSs. The authors of these works have usually assumed that the latent hardening exceeds the active one, and therefore when the deformation of a representative macrovolume takes place, an effective anisotropic YS that differs significantly from the von Mises sphere occurs. This effect manifests itself, for example, as an increase in the equivalent flow stress after the sample’s rotations are modeled using the ECAP technique [71,72].

In all the works cited above, the cross-hardening effect was investigated in the loading–unloading experiments. As noted above, this is due to the impossibility of experimentally realizing the stages of loading with SPCs that occur immediately one after another. However, in real technological processes, continuous loading with SPCs would most likely take place, and therefore it is essential to simulate loading with SPCs without unloading [17]. It is also reasonable to analyze the difference between the results of the simulation experiments with and without unloading. In particular, it would be worth considering the assumption that the effect of unloading on the stress–strain state should be taken into account only in the strain section that follows the strain-path change, which corresponds to the material memory trace [32,73]. The data given below confirm this assumption.

In this paper, a simple two-level statistical CM is presented for the description of the inelastic deformation of FCC polycrystals, where an intragranular dislocation slip is considered as the main mechanism of crystallite deformation at the mesolevel and the rotations of crystallite lattices are taken into account [18]. From a computational point of view, statistical models are much more efficient than self-consistent and direct models [8], which is why they are widely used to study the real technological processes of thermomechanical treatment.

It is worth noting that the authors of studies on the cross-hardening effect with the help of statistical [52,66,67] and self-consistent [47,68,70] constitutive models use special hardening laws to provide high latent hardening. In [26], the authors considered a self-consistent model with constant hardening matrix coefficients and noted that to correctly describe the cross-loading contraction and latent extension, it was necessary to write kinetic relations for the constant hardening coefficients. We demonstrate that the description of the cross-hardening effect is also possible with the hardening law widely used in CP [74]. The relations of the statistical constitutive model and the results of the identification of CMs for the polycrystalline aluminum samples are given in Section 2.

Section 3 presents the analysis of the results on kinematic loadings with SPCs (continuous and with unloading). It also contains the results of a statistical analysis of the motion of a mesostress in the stress space on the crystallite yield surface and a comparison of the obtained data with the data calculated using the isotropic hardening law for slip systems. According to the authors of this study, such analysis is important from a methodological point of view because it clarifies the capabilities of two-level CMs for describing complex loading.

## 2. Two-Level Statistical Constitutive Model for Describing Inelastic Deformation of FCC Polycrystals

We used a two-level statistical constitutive model for describing the inelastic deformation of FCC polycrystals. In models of this type, a representative volume of a polycrystal consisting of mesolevel elements (homogeneously deformed crystallites) is considered a macrolevel element [18,75,76,77]. Polycrystal stresses are obtained at the macrolevel from the averaging of the stress values of crystallites (hereinafter, the crystallite index is omitted):

(1) $$Κ=\langle \kappa \rangle ,$$

where Κ is the weighted Kirchhoff stress tensor at the macrolevel, $\kappa =\overset{ο}{\rho }/\hat{\rho }\sigma$ is the weighted Kirchhoff stress tensor at the mesolevel, σ is the Cauchy stress tensor at the mesolevel, $\overset{ο}{\rho },\hat{\rho }$ are the crystallite material densities in the initial (unloaded) and current configurations, and $\langle \cdot \rangle$ denotes the averaging procedure.

The system of equations used to describe the behavior of an individual crystallite is written as [18]:

(2) $$\kappa^{cor}\equiv \frac{d\kappa }{dt}+\kappa \cdot \omega -\omega \cdot \kappa =п:(l-\omega -\sum_{k=1}^{K}{\dot{\gamma }}_{\;}^{\left(k\right)}b^{\left(k\right)}n^{\left(k\right)}),\;{\dot{\gamma }}_{\;}^{\left(k\right)}={\dot{\gamma }}_{0}{\left(\frac{\tau^{\left(k\right)}}{\tau_{c}^{\left(k\right)}}\right)}^{m}H(\tau^{\left(k\right)}-\tau_{c}^{\left(k\right)}),\;\;\;\;\;\;\;\;\;\;\;\;\;\;\;\;\;\;\;\;k=1,\ldots ,K,\;\tau^{\left(k\right)}=b^{\left(k\right)}n^{\left(k\right)}:\kappa ,\;\;\;\;\;\;\;\;\;\;\;\;\;\;\;\;\;\;\;k=1,\ldots ,K,\;{\dot{\tau }}_{c}^{\left(k\right)}=F(\gamma^{\left(j\right)},{\dot{\gamma }}_{\;}^{\left(j\right)}),\;\;\;\;\;\;\;\;\;\;\;\;\;\;\;\;\;\;j,k=1,\ldots ,K,\;\omega =\omega (l,{\dot{\gamma }}_{\;}^{\left(k\right)}),\;\dot{ο}\cdot {ο}^{T}=\omega ,\;l=\hat{\nabla }v^{T}=\hat{\nabla }V^{T}=L,$$

where the upper index *cor* denotes the corotational derivative independent of the choice of a reference frame; п is the elastic property tensor, whose components are constant in the moving coordinate system, which rotates with a spin ω and specifies the (quasi) rigid motion (the corotational derivative) [78]; $l=\hat{\nabla }v^{T}$ is the velocity gradient; $b^{\left(k\right)}$ and $n^{\left(k\right)}$ are the unit vectors of the slip direction and the slip plane normal (in the current configuration) of edge dislocations; *K* is the doubled number of the crystallographic slip systems; ${\dot{\gamma }}_{\;}^{\left(k\right)}$ is the shear rate for the slip system *k*; ${\dot{\gamma }}_{0}^{\left(k\right)}$ is the shear rate for the slip system *k* in the case when the shear stress reaches its critical value; $\tau^{\left(k\right)}$ and $\tau_{c}^{\left(k\right)}$ are the shear and critical shear stresses for the slip system *k*; m is the strain rate sensitivity exponent of the material (in a dislocation slip mode); H(⋅) is the Heaviside function; F(⋅) is the function for calculating the rate of the critical shear stresses along the slip systems (hardening law); and ο is the tensor of the actual orientation of the moving coordinate system with respect to the fixed laboratory coordinate system (LCS). The last relation in (2) is Taylor’s iso-strain hypothesis. Note that instead of using the corotational derivative in relation (2)_1_, in some papers [79,80], the authors explicitly say that they use a linear constitutive relation written in the local coordinate system of crystallites.

In this study, we used two hardening laws (2)_4_:

- anisotropic hardening law [74,81]:
  (3) $${\dot{\tau }}_{c}^{\left(k\right)}=\sum_{l=1}^{K}h^{\left(kl\right)}|{\dot{\gamma }}^{\left(l\right)}|,\;k=1,\ldots ,K,\;h^{\left(kl\right)}=\left[q_{lat}+(1-q_{lat})\delta^{kl}\right]h^{\left(l\right)},\;h^{\left(l\right)}=h_{0}{\left|1-\tau_{c}^{\left(l\right)}/\tau_{sat}\right|}^{a},$$
- isotropic hardening law:

(4) $${\dot{\tau }}_{c}^{\left(k\right)}=AB{\dot{\Gamma }}^{B-1},\;k=1,\ldots ,K$$

where $h^{\left(kl\right)}$ is the matrix component characterizing the effect of the slip system *l* on the hardening along the slip system *k*, $q_{lat}$ is the latent hardening parameter, $\delta^{kl}$ is the Kronecker delta, $\tau_{sat}$ is the saturation stress, $h_{0}$ and a are the hardening law parameters (identified by fitting a model to the experimental data), A and B are the isotropic hardening law parameters, and $\dot{\Gamma }=\sum_{k=1}^{K}{\dot{\gamma }}^{\left(k\right)}$ is the integral estimate of the plastic shear rate. The description of the probable annihilation of the dislocations and reverse loadings at which the SS active during the first stage can be activated in the opposite direction was beyond the scope of this study. The considered anisotropic hardening law (3) has a simple mathematical formulation. Note that there are other methods for correctly describing the effects under complex loading with a strain-path change, for example, if we do not use the assumption of a doubled number of slip systems, which is computationally efficient, the model is complemented by equations to describe kinematic hardening [28]. In addition, it should be noted that for materials prone to twinning, the description of complex loadings with SPC will be even more difficult, since twinning significantly affects hardening and de-twinning and double-twinning processes are realized during the strain-path change [27], which must be taken into account in the model.

In some papers, the cross-hardening effect has been described using complex hardening laws. Under these laws, critical shear stresses on the slip systems are determined by the sum of the terms responsible for different hardening mechanisms. In [52], in contrast to (3), the influence of each active SS on the hardening along other SSs was not explicitly considered. For all SSs, it was assumed that the hardening depended on the total shear rate and the hardening coefficient was much larger for inactive slip systems, which allowed the implementation of “the extra latent hardening of non-active slip systems”. In [47], the authors proposed complex kinetic relations for individual terms determined by shear vectors to assess the interactions between the different slip systems. We have chosen the hardening law (3) for our study because it is easy to implement and due to its transparent physical meaning. This law is often used to simulate the deformation of aluminum samples under monotonic loading [82,83,84,85,86].

The results of the calculations obtained in our investigation by applying (3) indicate the possibility of a correct description of the cross-hardening effect when setting the value of the latent hardening parameter $q_{lat}=2$. Usually, in crystal plasticity, $q_{lat}=1.4$ is taken to demonstrate that, at active slips along some SSs, the obstacles for inactive SSs are created more intensively than those for this SS [18]. Note that the widely used value of the parameter $q_{lat}=1.4$ was obtained empirically for monotonic loadings. It is evident that for other values of $q_{lat}$, the remaining model parameters can be identified to match the experimental data. Generally speaking, researchers often report that the latent hardening coefficient (namely, at the initial stage of inelastic deformation), including under complex loading, can be observed in a wide range. The authors in [47] noted that “In any case, while one can be successful in modeling monotonic straining and texture evolution with any reasonable latent hardening scheme…, whether or not the evolution of latent hardening during straining is correctly captured is not apparent until there is a strain path change”. In [11], the authors investigated, together with the typical value of latent hardening parameter $q_{lat}=1.4$, the case $q_{lat}=3$, which enabled them to conclude that “the overall behavior of the models is quite similar”. Note that generally, it is quite reasonable to use more detailed hardening laws for describing the cross-hardening-type effects, for example, those that explicitly describe the evolution of the dislocation densities along slip systems and take into account the stacking fault energy. However, in this case, the CM was more complicated—it became a three-level constitutive model [87].

Most crystal plasticity studies describe the rotation of a crystallite lattice in the framework of the Taylor’s spin model [76]:

(5) $$\omega =\frac{1}{2}(l-l^{T})-\frac{1}{2}\sum_{k=1}^{K}{\dot{\gamma }}_{\;}^{\left(k\right)}(b^{\left(k\right)}n^{\left(k\right)}-n^{\left(k\right)}b^{\left(k\right)}).$$

The lattice rotation model [78,88] in which the moving coordinate system is associated with the symmetry elements of the crystal lattice, turned out to be more physically substantiated. It has previously been shown [89] that such models produce almost similar results, only slightly differing from those obtained with the model of the rotation determined by the orthogonal tensor in the polar decomposition of the elastic component of the deformation gradient [90]. The Taylor’s spin model was used as it is easier to implement.

We note that the rate form of the CM used in this paper in the current configuration provided results that were close to those obtained using the widely applied formulation in the unloaded configuration [11,81,90,91,92,93]. A comprehensive discussion of this phenomenon was given in [23,94], where the connection between these models was established by employing the formulation based on an explicit selection of a moving coordinate system in the multiplicative decomposition of the deformation gradient [78,88].

The model parameters were determined based on the experimental data for the tensile curve plotted for the aluminum samples [95].

The representative macrovolume of a material contains 343 crystallites, assuming that their initial orientation distribution is uniform. Table 1 contains the material parameters and hardening law parameters determined by the identification procedure.

**Figure 1:**
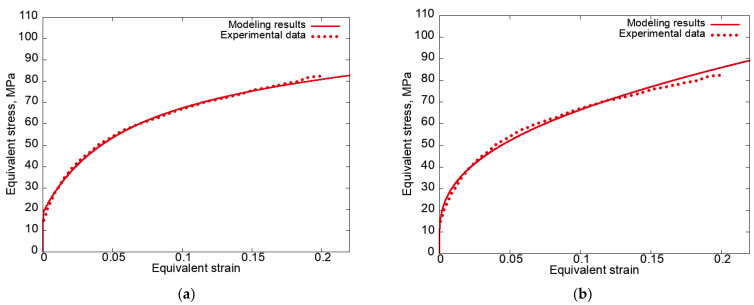
Equivalent stress–equivalent strain curves plotted using the simulation results for quasi-uniaxial tension along the Ox_1_ axis using (**a**) anisotropic hardening law, (**b**) isotropic hardening law, and the experimental results under uniaxial tension along the Ox_1_ axis [95].

We considered kinematic loading with the velocity gradient $L(t)=\dot{\varepsilon }p_{1}p_{1}-\frac{\dot{\varepsilon }}{2}p_{2}p_{2}-\frac{\dot{\varepsilon }}{2}p_{3}p_{3}$, where $p_{i},i=\bar{1,3}$ is the basic LCS and $\dot{\varepsilon }=0.0017$ s^−1^ is the strain rate. In [74], this loading was proposed for the statistical CM as an approximation of uniaxial tension—the calculated equivalent stress for the polycrystals turned out to be close to the uniaxial stresses in a full-scale experiment. For brevity, we called this kinematic loading “quasi-uniaxial tension”. Note that according to the numerical experiments, the activities of the slip systems under quasi-uniaxial tension and uniaxial tension were very close. This allowed for the use of quasi-uniaxial tension as an approximation of uniaxial tension. It is worth noting that, due to the initial isotropy of the sample, the same results were expected to be obtained under quasi-uniaxial tension along each of the three axes. Figure 1 shows the dependences of the equivalent stress $\sigma_{e}\left(t\right)=\sqrt{\frac{3}{2}S\left(t\right):S\left(t\right)}$ and $S\left(t\right)=dev\left(Κ\left(t\right)\right)$ on the equivalent strain [96,97,98] $\varepsilon_{e}\left(t\right)=\int_{0}^{t}\sqrt{\frac{2}{3}D\prime \left(t\right):D\prime \left(t\right)}dt$, $D\prime \left(t\right)=dev\left(D\left(t\right)\right)$ and $D\left(t\right)=\frac{1}{2}\left(L\left(t\right)+L^{T}\left(t\right)\right)$, calculated under quasi-uniaxial tension for the two hardening laws and under uniaxial tension along the Ox_1_ axis in an experiment [95].

Figure 1 shows that the simulation results are in satisfactory agreement with the experimental data [95] obtained under the considered deformations. Note that as the equivalent strain increased, the isotropic hardening law hardly corresponded to the experimental data since it did not take into account saturation during hardening. This law was used to perform the test calculations at small strains. To describe the real processes of thermomechanical treatment taking place at large strains, the anisotropic hardening law of type (3) or more complex ones should be used.

## 3. Results and Discussion

This section describes and analyzes some results of the modeling of the loading processes with strain-path changes using a two-level CM. We considered kinematic loadings that approximately describe the rolling and tension, simple shear, as well as the two-stage sequences of these processes. To determine the SPC angle, we used the measure proposed in the work [99]:

(6) $$\cos\phi_{D}=\frac{L_{1}:L_{2}}{\left\|L_{1}\right\|\left\|L_{2}\right\|},$$

where $\left\|A\right\|=\sqrt{A:A^{T}}$ is the tensor norm.

For the convenience of presenting the results, we describe the loading modes with the strain-path changes considered in this paper:

*(1) Tension–rolling* 

The comparison of the simulation results with the experimental data on loadings with SPCs was performed by observing quasi-uniaxial tension along the Ox_3_ axis and subsequent rolling along the Ox_1_ axis, which was modeled under tension along the Ox_1_ axis and under compression along the Ox_2_ axis. The approximation of the rolling process was made through kinematic loading with a velocity gradient $L(t)=\dot{\varepsilon }p_{1}p_{1}-\dot{\varepsilon }p_{2}p_{2}$. Note that the same approximation for the moderate deformations (prescribed via the deformation gradient) was used in [100].

*(2) Rolling–tension* 

Loadings were given as specified in mode 1 but in reverse order, i.e., at the first stage—rolling and at the second stage—quasi-uniaxial tension along the Ox_3_ axis.

*(3) Tension–shear* 

Quasi-uniaxial tension along the Ox_3_ axis and kinematic loading under simple shear with a velocity gradient $L(t)=\dot{\varepsilon }p_{1}p_{2}$ were considered.

*(4) Shear–tension* 

Loadings were given as specified in mode 3 but in reverse order, i.e., at the first stage—simple shear and at the second stage—quasi-uniaxial tension along the Ox_3_ axis.

For loading modes 1–4, the strain-path change angle (6) was $\phi_{D}=90{}^{\circ}$. Note that in all loading modes, the texture evolution was not considered since in the studied strain range it did form to an extent, which made it possible to reliably assess the difference in the uniform distribution of the orientations.

**Figure 2:**
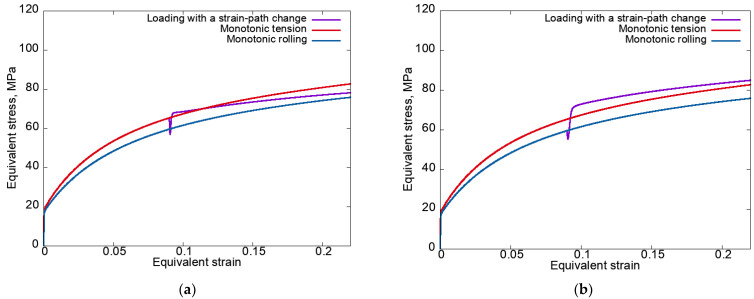
Equivalent stress–equivalent strain curves plotted for modes 1 (**a**) and 2 (**b**) using an anisotropic hardening law without unloading.

Figure 2 presents the equivalent stress–equivalent strain curves plotted according to modes 1 (at left) and 2 (at right) for the anisotropic hardening law (3) without unloading.

It can be seen that a local decrease in stress occurred in both cases. Under the loading conditions according to mode 2 (rolling–tension) after a local decrease in stress, the equivalent stress increased sharply and reached values higher than in the case of monotonic tension (the cross-hardening effect was realized). Under mode 1 (tension–rolling), the excess in the equivalent stress at the second stage (rolling) of the equivalent stress under monotonic tension (Figure 2a) is explained by the large SPC angle ($\phi_{D}=90{}^{\circ}$) at significant latent hardening ($q_{lat}=2$).

**Figure 3:**
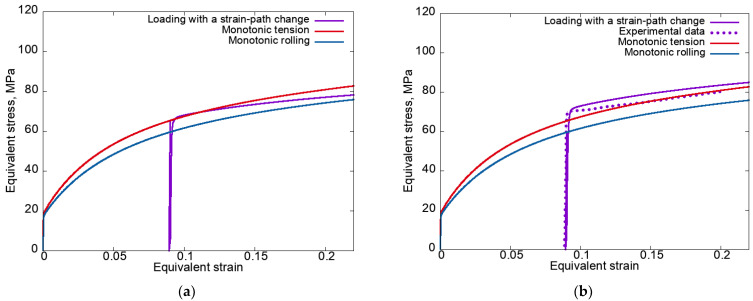
Equivalent stress–equivalent strain curves plotted for modes 1 (**a**) and 2 (**b**) using an anisotropic hardening law with unloading and the experimental data for uniaxial tension along the Ox_3_ axis upon the end of the pre-rolling stage [95].

In some cases, the experiments on loading with strain-path changes were carried out with unloading. Therefore, in order to compare the obtained numerical data and the existing experimental results, it was necessary to analyze the considered complex loadings, taking into account the unloading phase. Within the framework of the proposed model, the unloading was specified as the process of deformation. It continued until the equivalent macrostress became close (with a given error) to a value of zero under loading with a velocity gradient L(t)=−αΚ(t), where $\alpha =2\times 10^{-4}$ (MPa·s)^−1^. Figure 3 shows the equivalent stress–equivalent strain curves plotted for modes 1 and 2 using an anisotropic hardening law with unloading and the experimental data for uniaxial tension along the Ox_3_ axis upon the end of the pre-rolling stage [95].

It follows from Figure 3 that the results of modeling under loading with SPCs are in satisfactory agreement with the experimental data [95] on the level of stresses after the strain-path changes. Thus, the proposed statistical CM (1)–(2) is capable of describing the cross-hardening effect in the context of simple hardening laws. Note that the discrepancies between the obtained results and the results of the numerical experiments without unloading (Figure 2) were observed only near the break point of the strain path. It was shown that for the cases with and without unloading, there were insignificant differences in the implementation of shears along the SS. Note that these differences insignificantly affected the material response at the macrolevel. To describe the experimentally observed softening (Figure 3b) in terms of any model, this mechanism must be incorporated into the hardening law.

The two-level CP CM makes it possible to analyze how the IDS is realized in crystallites.

**Figure 4:**
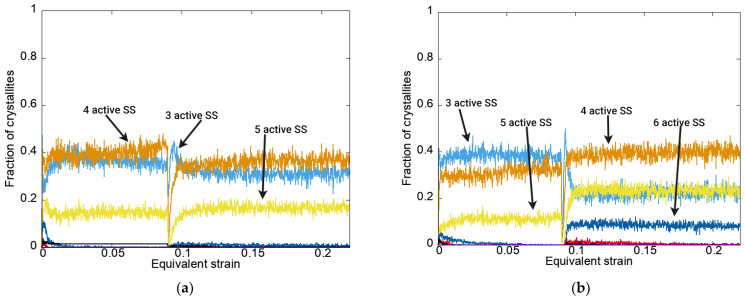
Dependences of the fraction of crystallites with a certain number of slip systems active according to the Schmid criterion on the equivalent strain for modes 1 (**a**) and 2 (**b**) using an anisotropic hardening law without unloading.

Figure 4 shows the dependences of the fraction of crystallites with a certain number of slip systems active according to the Schmid criterion, determined using the model describing the deformation processes under modes 1 and 2 on the equivalent strain. The jump-like dependences occurred due to the frequent change in the active SSs (no time-interval averaging).

The data given in Figure 4 demonstrate that under rolling (second load stage—mode 1, first load stage—mode 2), a smaller number of active SSs were realized compared to those that occurred under tension (first load stage—mode 1, second load stage—mode 2).

**Figure 5:**
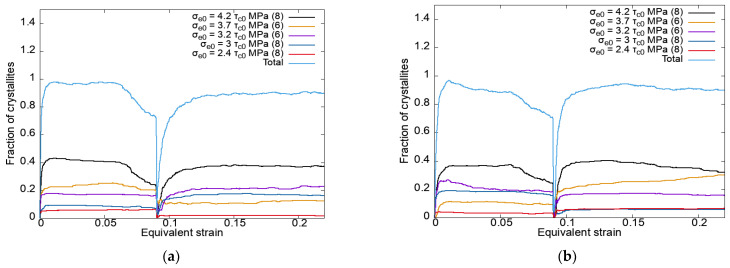
Dependences of the fraction of crystallites referring to specific classes (belonging was defined with a tolerance of 5 MPa) on the equivalent strain for modes 1 (**a**) and 2 (**b**) using an anisotropic hardening law without unloading.

Now, it is advantageous to analyze the statistical estimates of the location of the mesostress in the stress space on the crystallite yield surface. An assessment of the proximity of the mesostress in the stress space to the vertex of the yield surface of a particular class was determined by the difference between the shear stresses on the slip system and their critical values: if the tolerance deviation did not exceed 5 MPa (as specified in this study) for all systems, the intersection of which forms the vertex, then the mesostress in the stress space was considered to be close to the vertex of this class. As is known, when using the Schmid criterion for an FCC lattice, there are five classes of high-order vertices of the initial YS corresponding to the different values of the equivalent stress [101,102]. Figure 5 gives the dependences of the fraction of crystallites and the equivalent mesostresses in the stress space, which are close to the vertices of specific classes, on the equivalent strain for the given loading modes using an anisotropic hardening law without unloading. The order of the yield surface vertex is given in parentheses, indicating the number of the likely active slip systems.

**Figure 6:**
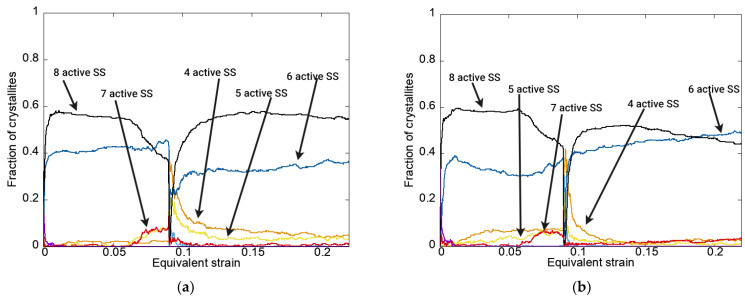
Dependences of the fraction of crystallites with a specified number of likely active slip systems (determined with a tolerance of 5 MPa) on the equivalent strain for modes 1 (**a**) and 2 (**b**) using an anisotropic hardening law without unloading.

It should be noted that the decrease in the number of grains with eight likely active slip systems after the strain had attained a certain value is logical—the YS was transformed in such a way that the eighth-order vertices ceased to exist due to anisotropic hardening. This was confirmed by the increase in the fraction of crystallites with seven likely active slip systems (Figure 6) already under first-stage monotonic loading.

Analysis of the results given in Figure 5 indicates that, under mode 1 (tensile–rolling) at the second loading stage, the crystallite distribution over the equivalent stress classes occurred in such a way that the fraction of crystallites with high equivalent stress decreased, and under mode 2 (rolling–tension) increased. The data illustrating such behavior of the mesostress in the stress space on the YS explain the increase/decrease in the macrolevel equivalent stresses observed in Figure 2.

**Figure 7:**
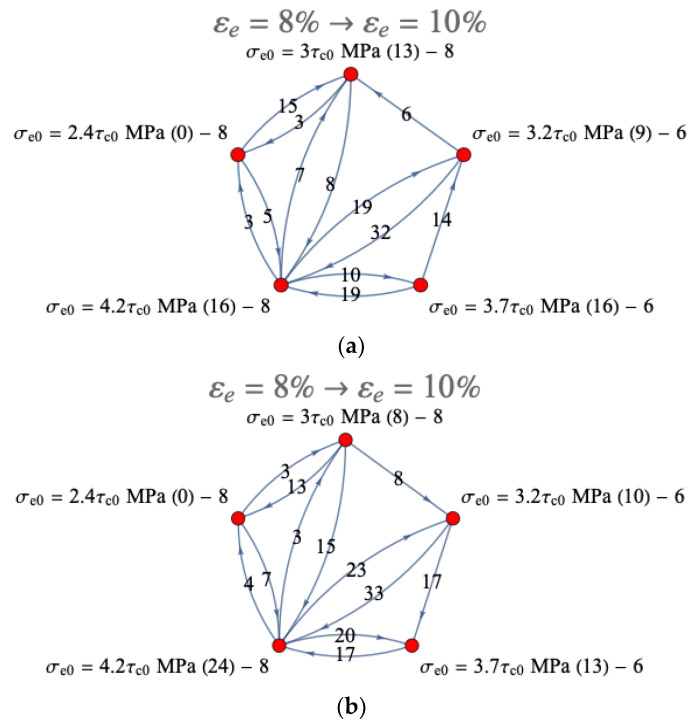
Graph representing transitions of the mesostress in the stress space from the yield surface vertices at the initial and final states, corresponding to 8% and 10% equivalent strains, respectively, for modes 1 (**a**) and 2 (**b**) using an anisotropic hardening law without unloading (the number of mesostresses in the stress space that have passed via the transitions is shown on the edges, and the number of mesostresses in the stress space that remain in their classes in the considered strain range is shown in brackets at the graph vertices; the last digit characterizes the number of likely active slip systems in this class).

In order to visualize the transitions of the mesostress in the stress space from one class of yield surface vertices to another, the corresponding graphs were plotted. To this end, the distribution of the mesostress in the stress space over the yield surface vertices at an 8% equivalent strain was taken as the initial state, and the distribution of the mesostress in the stress space at a 10% equivalent strain was taken as the final state. The mesostress in the stress space was considered to have passed from one class of vertices to another if it belonged to different classes at the indicated instants of strain; otherwise, the mesostress in the stress space was assumed to remain in its class. Figure 7 shows the graphs of the mesostresses in the stress space transitions from the yield surface vertices at the initial state corresponding to an 8% equivalent strain and at the final state corresponding to a 10% equivalent strain for the considered loading modes using an anisotropic hardening law without unloading. The number of mesostresses in the stress space that have passed via the transitions is indicated on the edges, and the number of mesostresses in the stress space remaining in their classes in the considered range of strain is given in brackets at the graph vertices; the last digit characterizes the number of likely active slip systems in this class.

In the above graph, we can see that under mode 1 (tension–rolling), the number of mesostresses in the stress space transferred to classes with high equivalent stress was less than those under mode 2 (rolling–tension). This confirms that during tension, the mesostresses in the stress space tended to approach the vertices with higher equivalent stress, whereas during rolling the lower equivalent stress on the yield surface, in turn, led to an increase/decrease in the equivalent stresses (Figure 2). The presented results are in good agreement with the data shown in Figure 5.

For completeness analysis, we considered loading modes 1 and 2 using an isotropic hardening law (4).

**Figure 8:**
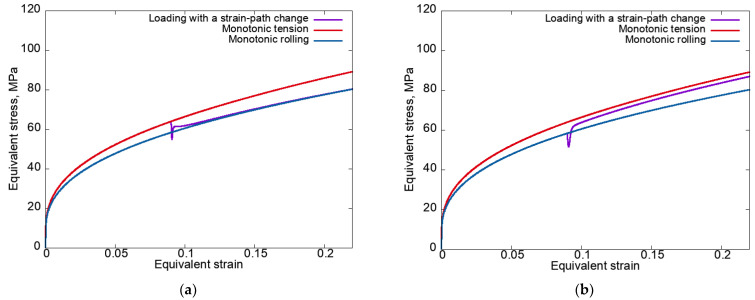
Equivalent stress–equivalent strain curves plotted for modes 1 (**a**) and 2 (**b**) using an isotropic hardening law without unloading.

Figure 8 shows the equivalent stress–equivalent strain curves plotted for modes 1 and 2 using an isotropic hardening law without unloading.

It can be seen in Figure 8a that in contrast to an anisotropic hardening law when using an isotropic law, the curve for loading mode 1 (tension–rolling) at the second strain path almost immediately after the SPC coincided with the monotonic loading curve, which indicates the fulfillment of the principle of fading memory. The non-fulfillment of this principle for mode 2 (Figure 8b) is due to the fact that during the first stage of loading (rolling), the anisotropy determined by the texture was more pronounced. When the rotation mechanism was turned off and a larger range of deformation was considered, the curve for the second stage of mode 2 also practically coincided with the curve for monotonic loading. Note that the differences in the way the stress–strain curves approached (after the SPC) the corresponding monotonic curves (from above for mode 1, from below for mode 2), as shown in Figure 8, are due to the previous loading–loading at the first stage being carried out with higher equivalent stress, and then the curves started to approach from above, and vice versa.

In the case of an anisotropic hardening law, the effect of increasing the equivalent stresses (Figure 2) was enhanced due to the presence of latent hardening along the slip systems that were inactive at the first stage.

For the case of isotropic hardening under the same impacts, a numerical experiment with unloading was performed, and the behavior of the mesostress in the stress space on the yield surface of the crystallites was analyzed. The obtained results (including data on the activity of slip systems for all modes) are in qualitative agreement with the corresponding results for the anisotropic hardening law and they are not presented for the sake of brevity.

For loading modes 3 and 4, only the stress–strain curves for the loadings with the SPC using anisotropic and isotropic hardening laws without unloading are given below since the results of the statistical analysis of the behavior of the mesostress in the stress space on the yield surface of the crystallites qualitatively correspond to the results for loading modes 1 and 2.

**Figure 9:**
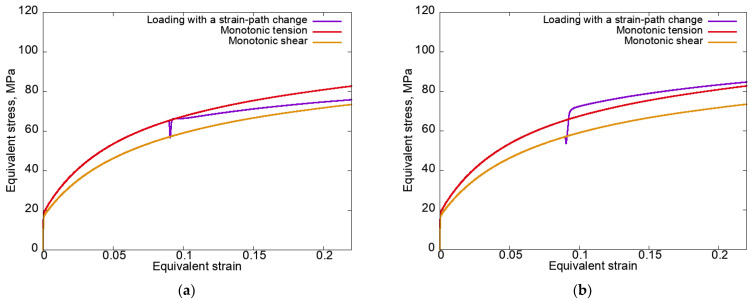
Equivalent stress–equivalent strain curves plotted for loading modes 3 (**a**) and 4 (**b**) using an anisotropic hardening law without unloading.

Figure 9 shows the equivalent stress–equivalent strain curves plotted for loading modes 3 and 4 using an anisotropic hardening law without unloading.

The difference between the monotonic curves for quasi-uniaxial tension and simple shear is consistent with the experimental data for aluminum alloy AA6061 [17].

We note that in [17], the authors analyzed in detail the change in the yield surface using the CP FEM for different loadings; however, not much attention was paid to studying the behavior of the mesostress in the stress space on the yield surface for the crystallites that make up the macroscale RV. In this paper, we considered a two-level statistical CM to carry out a statistical analysis of the behavior of the mesostress in the stress space on the yield surface of crystallites, which is important for understanding the capabilities of CP CM.

The considered effects manifested themselves in an analogous way in the case of isotropic hardening (4).

**Figure 10:**
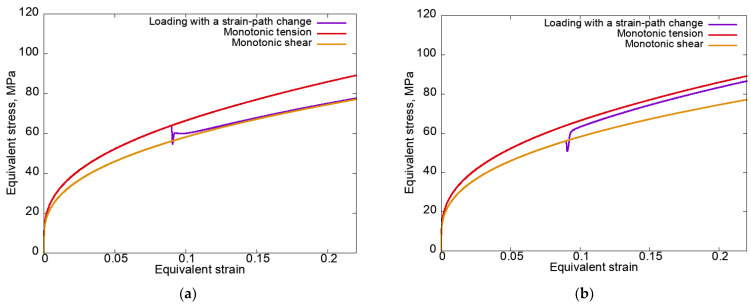
Equivalent stress–equivalent strain curves plotted for loading modes 3 (**a**) and 4 (**b**) using an isotropic hardening law without unloading.

Figure 10 presents the equivalent stress–equivalent strain curves plotted for loading modes 3 and 4 using an isotropic hardening law without unloading.

It was observed that the equivalent stress at the final stage under mode 4 (shear–tension) was significantly higher than that observed under mode 3 (tension–shear). These results are consistent with the experimental data on the evolution of the yield surface obtained in [17] for an aluminum alloy AA606.

Based on the above analysis, we can conclude that the differences in the behavior of the mesostress in the stress space on the yield surface of the crystallites for modes 1 and 2 (3, 4) were caused by the differences in the evolution of the internal defect structure under different loadings. This, in turn, led to “transitions” between the stress–strain curves for the given loadings. If we consider this issue in the context of the motion of the mesostress in the stress space on the yield surface, then it can be noted that generally, the yield surface of a crystallite has a complex shape and depending on the specified rigid loading, the mesostress in the stress space tended to fall on the faces and edges of the yield surface, as well as at its vertices with different equivalent stress.

## 4. Conclusions

In this study, we demonstrated the potential of a two-level statistical constitutive model for describing the behavior of FCC polycrystals. The identification of the model parameters for polycrystalline aluminum was carried out based on the experimental data for monotonic tension and complex loading with strain-path changes. The behavior of the mesostress in the stress space on the yield surface of the crystallites was comprehensively analyzed. It was found that the effects of the increases/decreases in the stresses at a change in the strain path were associated with the characteristic transitions of the mesostress in the stress space between the yield surface vertices at the mesolevel. The obtained results are in satisfactory agreement with the known data, meaning that the yield surface of polycrystals is anisotropic and transforms in a complex way. At the same time, the development of a universal scalar measure of accumulated plastic strain, which could be used to introduce the generalized single curve hypothesis (at least for monotonic loading of materials with cubic symmetry and initially uniform distribution of grain orientations) is an important factor in solving the problems of technological plasticity. A possible solution is to apply the scalar characteristics (for example, averaged accumulated shears) of deformation mechanisms at lower scale levels, which implies the use of multilevel constitutive models [103].

Thus, it is shown that the basic two-level constitutive crystal plasticity models are able to describe experimentally observed effects under moderate strains and analyze complex loading processes, which allows one to use them as a basis for extended models. These models can be complicated by considering large deformation gradients (taking into account texture) as well as by describing other mechanisms (refinement, recrystallization, fracture, etc.). Many research teams, including the authors of this paper, are involved in studying these issues.

## Figures and Tables

**Table 1:** Parameters of CM for the aluminum.

Parameter	Definition	Value
${п}_{1111}$	independent components of the elastic property tensor [77]	106.75 GPa
${п}_{1122}$		60.41 GPa
${п}_{1212}$		28.34 GPa
${\dot{\gamma }}_{0}$	parameters for viscoplastic relation (2)_2_ [77]	0.001 s^−1^
m		50
$\tau_{c0}$	initial critical shear stress for the anisotropic hardening law	6 MPa
$q_{lat}$	latent hardening parameter	2
$\tau_{sat}$	saturation stress	34 MPa
$h_{0}$	anisotropic hardening law parameters	115 MPa
a		2.25
$\tau_{c0}$	initial critical shear stress for the isotropic hardening law	3 MPa
A	isotropic hardening law parameters	29 MPa
B		0.4

## Data Availability

The data presented in this study are available on request from the corresponding author.
